# NCT-CXR: Enhancing Pulmonary Abnormality Segmentation on Chest X-Rays Using Improved Coordinate Geometric Transformations

**DOI:** 10.3390/jimaging11060186

**Published:** 2025-06-05

**Authors:** Abu Salam, Pulung Nurtantio Andono, Purwanto, Moch Arief Soeleman, Mohamad Sidiq, Farrikh Alzami, Ika Novita Dewi, Suryanti, Eko Adhi Pangarsa, Daniel Rizky, Budi Setiawan, Damai Santosa, Antonius Gunawan Santoso, Farid Che Ghazali, Eko Supriyanto

**Affiliations:** 1 Faculty of Computer Science, Universitas Dian Nuswantoro, Semarang 50131, Indonesia; pulung@dsn.dinus.ac.id (P.N.A.);; 2 Dinus Research Group for AI in Medical Science (DREAMS), Universitas Dian Nuswantoro, Semarang 50131, Indonesia; 3 OncoDoc AI Research Laboratory, Semarang 50131, Indonesia; 4 Faculty of Medicine, Universitas Dian Nuswantoro, Semarang 50131, Indonesia; 5 Division of Hematology-Medical Oncology, Faculty of Medicine, Diponegoro University, Dr. Kariadi General Hospital, Semarang 50244, Indonesia; 6 Radiology Department, Faculty of Medicine, Diponegoro University, Dr. Kariadi General Hospital, Semarang 50244, Indonesia; 7 Faculty of Dentistry, Lincoln University College Malaysia, SS6/12, Kelana Jaya, Petaling Jaya 47301, Malaysia; 8 Faculty of Electrical Engineering, Universiti Teknologi Malaysia, Johor Bahru 81310, Malaysia

**Keywords:** chest X-ray, pulmonary abnormalities, coordinate transformation, data augmentation, geometric transformation, multi-label segmentation

## Abstract

Medical image segmentation, especially in chest X-ray (CXR) analysis, encounters substantial problems such as class imbalance, annotation inconsistencies, and the necessity for accurate pathological region identification. This research aims to improve the precision and clinical reliability of pulmonary abnormality segmentation by developing NCT-CXR, a framework that combines anatomically constrained data augmentation with expert-guided annotation refinement. NCT-CXR applies carefully calibrated discrete-angle rotations (±5°, ±10°) and intensity-based augmentations to enrich training data while preserving spatial and anatomical integrity. To address label noise in the NIH Chest X-ray dataset, we further introduce a clinically validated annotation refinement pipeline using the OncoDocAI platform, resulting in multi-label pixel-level segmentation masks for nine thoracic conditions. YOLOv8 was selected as the segmentation backbone due to its architectural efficiency, speed, and high spatial accuracy. Experimental results show that NCT-CXR significantly improves segmentation precision, especially for pneumothorax (0.829 and 0.804 for ±5° and ±10°, respectively). Non-parametric statistical testing (Kruskal–Wallis, H = 14.874, *p* = 0.0019) and post hoc Nemenyi analysis (*p* = 0.0138 and *p* = 0.0056) confirm the superiority of discrete-angle augmentation over mixed strategies. These findings underscore the importance of clinically constrained augmentation and high-quality annotation in building robust segmentation models. NCT-CXR offers a practical, high-performance solution for integrating deep learning into radiological workflows.

## 1. Introduction

Medical imaging, particularly chest X-rays (CXR), is fundamental to global healthcare, serving as a primary tool for diagnosing and managing various pulmonary conditions. Precise and reliable segmentation of pulmonary abnormalities in CXR images is essential for accurate diagnosis, effective treatment planning, and continuous disease monitoring. However, semantic segmentation in chest X-rays remains a challenging task due to three major issues: (i) class imbalance, where certain pathologies such as pneumothorax or nodule are underrepresented, leading to biased model learning; (ii) annotation inconsistencies, as CXR labels are often generated through natural language processing or non-expert interpretation; and (iii) complex anatomical characteristics such as overlapping structures, irregular boundaries, and subtle textural differences.

The increasing volume of radiological examinations, coupled with a global shortage of radiologists, particularly in resource-constrained settings, exacerbates the challenges of pulmonary image segmentation. This shortage not only widens disparities in access to imaging services but also contributes to delays in diagnosis and treatment, negatively impacting patient outcomes [1,2,3]. Given these challenges, automated image analysis systems have emerged as a crucial solution. Deep learning has emerged as a powerful tool for medical image analysis and holds great potential for automating CXR interpretation [4,5,6]. However, its application to CXR segmentation is constrained by the quality of existing datasets and augmentation strategies. Widely used datasets such as NIH Chest X-ray often contain only image-level labels and suffer from high label noise [7]. Prior research typically focuses on limited pathologies [8,9], such as Abedalla’s study of pneumothorax detection [10], Zou’s work on cardiomegaly [11], and Kumarasinghe et al.’s exploration of pneumonia and COVID-19 [12]. Arora et al. examined a combination of four pathologies [13], yet comprehensive segmentation involving multiple concurrent thoracic conditions remains underexplored due to the absence of reliable pixel-level annotations.

To mitigate class imbalance and limited training data [14,15,16,17,18], various augmentation techniques, including rotation, flipping, scaling [19,20], and GANs [19,21,22], have been employed. However, many of these approaches fail to account for anatomical plausibility. Uncontrolled transformations can distort lung orientation [10,23], displace pathological regions, or introduce unrealistic features. For example, geometric augmentations applied without clinical constraints may degrade model performance by compromising spatial and diagnostic integrity. Although advanced architectures such as Kronecker convolutional networks [24] and domain adaptation techniques attempt to address data limitations, they often require complex training procedures and are rarely guided by domain-specific (clinical) constraints. As Pati et al. [25] demonstrated, inter-annotator variability can significantly impact segmentation labels, a problem also observed by Zhang et al. [26].

In this research, we utilize YOLOv8 for semantic segmentation due to its strong balance of real-time performance, architectural efficiency, and high segmentation accuracy. YOLOv8 extends the YOLO family [27,28,29,30] with advanced features such as decoupled head design, improved anchor-free detection, and native support for segmentation tasks. Its single-stage pipeline offers superior inference speed compared to encoder–decoder-based models (e.g., U-Net or DeepLabv3), making it well-suited for applications requiring low latency and clinical integration. Unlike many standard segmentation architectures, YOLOv8 is particularly efficient in scenarios where bounding box and mask predictions are tightly correlated, such as thoracic abnormalities in chest X-rays.

The aim of this research is to improve the accuracy and anatomical reliability of deep learning-based pulmonary segmentation in chest X-rays through a combined augmentation and annotation refinement framework, termed NCT-CXR. NCT-CXR introduces a set of precisely calibrated coordinate-aware transformations, including small-angle discrete rotations (±5°, ±10°) and intensity augmentations, each designed to preserve anatomical structure while enhancing model generalizability [10,23,31]. Unlike many augmentation studies that apply large, randomized, or anatomically implausible transformations, this research proposes a clinically informed augmentation strategy, where each transformation is carefully selected and validated through expert consultation to maintain diagnostic relevance. The integration of radiologist-refined, multi-label pixel-level annotations with anatomically constrained, coordinate-level augmentation directly addresses the dual challenges of label noise and spatial distortion, both of which are under-explored in prior work. These augmentations are applied during training, not inference, to systematically improve model robustness against positional and acquisition variability commonly observed in real-world CXR data.

In addition to augmentation, this research contributes to a rigorous expert annotation refinement process to enhance the NIH Chest X-ray dataset. Original image-level labels are transformed into pixel-level, multi-label segmentation masks for nine thoracic pathologies using the OncoDocAI platform and dual-radiologist review. This addresses the critical limitation of noisy or ambiguous labels, yielding a higher quality training set for evaluating semantic segmentation models under clinically realistic conditions.

The aim of this research is to develop and evaluate an augmentation and annotation framework (NCT-CXR) that improves the accuracy and clinical reliability of deep learning-based pulmonary abnormality segmentation in chest X-ray images. The main contributions of this work are as follows:

– NCT-CXR framework: We propose a novel augmentation strategy that combines discrete-angle coordinate transformations (±5°, ±10°) with intensity-based modifications, optimized to preserve anatomical integrity during semantic segmentation.
– Expert annotation refinement: We introduce a rigorous annotation process involving dual radiologist review and the OncoDocAI platform to generate clinically validated, multi-label segmentation masks, addressing the known label noise in the NIH Chest X-ray dataset.
– Empirical evaluation with YOLOv8: We implement NCT-CXR on the YOLOv8 architecture and evaluate its effectiveness across four augmentation models using statistical validation (Kruskal–Wallis and Nemenyi tests).
– Improved performance for clinically relevant cases: Our results show significant gains in segmentation precision, particularly for pneumothorax, demonstrating the clinical potential of anatomically constrained augmentation.

The remainder of this paper is organized as follows: Section 2 reviews related literature on deep learning and augmentation in CXR segmentation; Section 3 outlines the NCT-CXR methodology; Section 4 presents experimental results; Section 5 discusses clinical implications and limitations; and Section 6 concludes the study with key insights and future directions.

## 2. Related Works

Recent advances in deep learning for medical image analysis have significantly propelled the field of automated chest X-ray interpretation. These advancements encompass architectural innovations, data handling strategies, and evolving semantic segmentation techniques [1,5,14,20]. The shift towards semantic segmentation, particularly in medical imaging, allows for more granular and clinically relevant analysis compared to traditional classification approaches [9,20,25]. This section examines pertinent literature across three key areas: deep learning architectures for medical image segmentation, data augmentation strategies in medical imaging, and methods for addressing class imbalance in medical datasets.

### 2.1. Deep Learning Architectures for Medical Image Segmentation

The evolution of deep learning architectures for medical image segmentation has witnessed significant progress, with YOLO-based architectures emerging as powerful tools. While traditional semantic segmentation networks like U-Net [32] have been widely adopted in medical imaging, YOLO-based architectures have gained traction due to their ability to efficiently combine object detection and segmentation [27,28,29,30]. The introduction of YOLOv8 [28] brought substantial improvements in segmentation capabilities, particularly in complex medical imaging tasks. Several studies have demonstrated the effectiveness of YOLO-based architectures in chest X-ray analysis, including the detection of COVID-19 pneumonia, where YOLO models effectively locate and segment the thoracic and lung regions [33,34]. These examples highlight the ability of YOLO-based models to handle multiple pathological conditions concurrently while maintaining computational efficiency. However, challenges remain in maintaining consistent performance across diverse pathologies, especially for rare conditions with limited training examples [5].

### 2.2. Data Augmentation Strategies in Medical Imaging

Data augmentation strategies in medical imaging have progressed beyond basic geometric transformations to encompass sophisticated techniques that preserve clinical validity. Recent research has explored various augmentation methods tailored to chest X-rays, ranging from conventional geometric transformations to advanced intensity-based modifications [19,20]. While traditional data augmentation methods like rotation and scaling have proven effective in general computer vision, their application in medical imaging demands careful consideration of anatomical constraints [10,23]. Researchers have investigated the impact of different augmentation strategies on model performance, emphasizing the preservation of diagnostic features [19]. Intensity-based augmentations, such as contrast adjustment and noise addition, have been shown to improve model robustness to variations in image acquisition conditions [21,35,36]. However, determining optimal augmentation parameters that enhance performance without introducing artifacts that compromise diagnostic accuracy remains a challenge. For example, excessive rotation can obscure anatomical structures, while insufficient augmentation may not provide sufficient variability for effective learning [37].

### 2.3. Addressing Class Imbalance in Medical Datasets

The challenge of class imbalance in medical imaging datasets has garnered considerable attention, particularly in chest X-ray analysis. Traditional approaches like oversampling and under sampling have shown limited success in medical imaging due to the complexity of pathological features [38]. Recent studies have explored more sophisticated methods, combining augmentation strategies with selective sampling techniques to address both class imbalance and annotation quality issues [15,20,39]. A crucial aspect often overlooked is the discrepancy between automated labels and expert annotations, which can significantly affect model performance [25]. The NIH Chest X-ray dataset [7], while widely used, exhibits this issue. Although image-level labels are provided, derived from text-mining radiological reports with an expected accuracy exceeding 90%, manual annotation remains necessary. This has spurred research into reconciling automated labels with expert annotations, especially in semantic segmentation.

While existing research has made progress in addressing individual challenges, a gap remains in integrating multiple approaches to simultaneously address class imbalance, annotation quality, and segmentation accuracy. Previous studies have often focused on either augmentation strategies [15,21] or architectural improvements [8,38], with limited attention to their systematic combination and rigorous statistical validation [10,35]. The impact of different augmentation strategies on semantic segmentation performance, particularly in the context of multiple co-existing pathologies, requires further investigation. Furthermore, while coordinate transformation in image augmentation has been explored [40,41,42], its specific application to semantic segmentation of chest X-rays, especially for multiple pathological regions, warrants more comprehensive research. This research addresses these gaps by proposing NCT-CXR, an integrated framework combining carefully calibrated augmentation strategies with YOLOv8’s semantic segmentation capabilities, supported by rigorous statistical analysis to validate the effectiveness of different augmentation combinations.

## 3. Methodology

**Figure 1:**
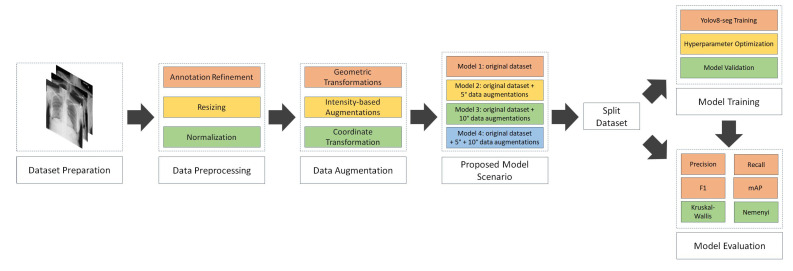
NCT-CXR model scenario.

This section outlines the proposed NCT-CXR framework, which integrates expert-guided annotation refinement, anatomically constrained data augmentation, and deep learning-based semantic segmentation using the YOLOv8 architecture. The methodology is organized into four subsections: dataset preparation, augmentation strategy, model training with hyperparameter optimization, and performance evaluation protocol. A visual summary of the NCT-CXR pipeline, from dataset preparation through augmentation, model training, and evaluation, is illustrated in Figure 1.

### 3.1. Dataset Preparation and Annotation Refinement

The experimental dataset was derived from the publicly available NIH Chest X-ray repository. A subset of 1061 images was selected to represent 9 clinically relevant thoracic abnormalities, in addition to normal findings. The distribution of cases across training and validation splits, organized by pathology type and folder index, is summarized in Table 1.

Given the limitations of the original dataset, particularly the presence of weak and noisy image-level labels, a refinement protocol was introduced to generate reliable pixel-level ground truth annotations. The annotation refinement process employed the OncoDocAI platform and involved a dual radiologist review. Two certified radiologists independently examined each image and collaboratively produced segmentation masks indicating the presence and boundaries of pulmonary abnormalities. This expert-led protocol ensured consistency across samples and produced clinically validated, multi-label masks that capture real-world co-occurrence of thoracic conditions. These refined masks were essential for training and evaluating the semantic segmentation framework.

All images were preprocessed by resizing them to a uniform dimension of 800 by 800 pixels using bilinear interpolation. This resolution was selected to maintain anatomical fidelity while ensuring compatibility with the input constraints of the YOLOv8 model. Pixel intensity normalization was performed using min–max scaling to map grayscale values into the [0, 1] range [43,44]. This normalization step facilitated model convergence and stabilized the training process.

### 3.2. Augmentation Strategy and Anatomical Constraints

To address the issue of class imbalance and improve model generalization, a clinically constrained augmentation strategy was implemented. Unlike conventional augmentation approaches that apply transformations arbitrarily, NCT-CXR focuses on preserving anatomical validity and spatial alignment between pulmonary structures and their corresponding segmentation masks. The geometric transformations employed include coordinate-aware discrete-angle rotations of ±5° and ±10°, selected through consultation with expert radiologists to reflect realistic patient positioning variations during imaging. These small-angle transformations preserve the topological integrity of thoracic anatomy while introducing controlled spatial variability essential for generalization.

Additional geometric augmentations include translation and scaling, applied within strict clinical thresholds: translations were constrained to a maximum of 2% of the image width or height, and scaling was limited to a 0.95–1.05 factor range. These constraints prevent distortion of lung morphology and displacement of pathological regions that could undermine diagnostic accuracy.

To simulate variations in acquisition conditions, intensity-based augmentations were also employed. Specifically, image brightness and contrast were adjusted randomly within ±10% of their original values, and Gaussian noise with variance sampled between 10.0 and 50.0 was injected to simulate radiographic artifacts. Crucially, all augmentation steps were performed using a coordinate-aware mapping framework, ensuring that the same transformations applied to the input image were also applied to the corresponding segmentation mask. This preserves pixel-level alignment and ensures clinical realism throughout the augmented dataset. This clinically grounded, multi-modal augmentation design distinguishes NCT-CXR from traditional pipelines that apply augmentations without medical oversight or spatial coherence, often introducing unrealistic variations. The structured augmentation approach employed in this study is both practical and novel in its explicit focus on preserving anatomical fidelity during data transformation.

In order to simulate clinically relevant variations in patient positioning while preserving anatomical consistency between chest X-ray images and their corresponding masks, the NCT-CXR framework incorporates a set of spatial and intensity transformations rooted in geometric operations. One of the primary transformations employed involves a rotation centered around the origin. The rotation of a pixel at coordinates (x, y) by an angle θ about the origin is represented using the transformation matrix in Equation (1). This rotation matrix preserves spatial relationships within the anatomical structures while allowing for controlled perturbations during training.

(1) $$\left[\begin{array}{c} x^{\prime }\\ y^{\prime } \end{array}\right]=\left[\begin{array}{cc} cos\theta & -sin\theta \\ sin\theta & cos\theta \end{array}\right]\left[\begin{array}{c} x\\ y \end{array}\right]$$

To introduce slight morphological variability, uniform scaling is applied along both the horizontal and vertical axes. The pixel coordinates are proportionally adjusted based on scaling factors sx and sy, which are selected within a constrained range, typically between 0.95 and 1.05, as shown in Equation (2). This controlled variation helps the model generalize to naturally occurring anatomical differences without introducing unrealistic distortions.

(2) $$S\left(x,y\right)=\left[\begin{array}{cc} s_{x} & 0\\ 0 & s_{y} \end{array}\right]\left[\begin{array}{c} x\\ yx \end{array}\right]$$

In terms of pixel intensity, each input image undergoes normalization to a fixed range of [0, 1]. This is achieved through a min–max normalization process that linearly rescales each pixel value relative to the minimum and maximum intensity levels present in the original image, as mathematically shown in Equation (3). This step is essential to ensure uniformity across the dataset, facilitating faster convergence and more stable training behavior.

(3) $$\begin{array}{c} x_{norm}=\frac{x-x_{min}}{x_{max}-x_{min}} \end{array}$$

(4) $$\begin{array}{c} y_{norm}=\frac{y-y_{min}}{y_{max}-y_{min}} \end{array}$$

All geometric transformations, such as rotation and scaling, are composed into a single affine mapping to streamline processing, as calculated in Equation (5). This composite transformation is applied uniformly to both the input image and its corresponding mask, thereby preserving pixel-wise alignment and ensuring accurate supervision during segmentation training.

(5) $$T=\left[\begin{array}{ccc} cos\theta & -sin\theta & t_{x}\\ sin\theta & cos\theta & t_{y}\\ 0 & 0 & 1 \end{array}\right]\left[\begin{array}{ccc} s_{x} & 0 & 0\\ 0 & s_{y} & 0\\ 0 & 0 & 1 \end{array}\right]$$

Finally, the transformation accuracy is optimized using a loss function that minimizes spatial distortion:

(6) $$L_{spatial}=\sum_{i=1}^{n}{∥\hat{P_{l}}-T\left(P_{i}\right)∥}_{2}+\lambda {∥T∥}_{F}$$

where $\hat{P_{l}}$ represents the target coordinates, $T(P_{i})$ are the transformed coordinates, ||.|| is the Frobenius norm, and λ is a regularization parameter (set to 0.01 in our implementation).

To provide a clear and reproducible understanding of the data augmentation process within the NCT-CXR framework, Algorithm 1 outlines the complete pipeline, from initial data loading to the generation of augmented image–label pairs. The algorithm is designed to preserve anatomical accuracy during augmentation by applying coordinate-aware geometric and intensity-based transformations. It ensures spatial alignment between original and transformed segmentation masks by integrating a coordinate transformation and normalization mechanism. The algorithm accepts directories of input images and their corresponding labels, defines output storage locations, and specifies the number of augmented samples to generate per original image. Transformation parameters, including rotation, scaling, shifting, brightness, contrast, and noise, are explicitly controlled to ensure clinically plausible modifications. Each step is structured to maintain consistency across the dataset and uphold the clinical validity of the augmented samples.
**Algorithm 1** Proposed Framework for Image Segmentation Data Augmentation**Require:**  **Input**  Original image directory, $Dir_{img}$;  Original label directory, $Dir_{lbl}$;  Output image directory, $Dir_{out_{img}}$;  Output label directory, $Dir_{out_{lbl}}$;  Number of augmentations per image, *N*;  Set of transformation parameters, $T=t_{1},t_{2},\ldots ,t_{n}$:   rotation limit: α;    shift limit: β;    scale limit: γ   brightness limit: δ;    contrast limit: ε;    noise variance limit: ς  **Ensure**:/* step: load and validate data */validate $Dir_{img}$ and $Dir_{lbl}$ existcreate $Dir_{out_{img}}$ and $Dir_{out_{lbl}}$ if not existget list of image paths *I* from $Dir_{img}$get list of label paths *L* from $Dir_{lbl}$/* step: process each image-label pair */**for** each image path *i* in *I* **do**:  read image img from *i*  get corresponding label path *l* from *L*  load segmentation labels *S* from *l*  get image dimensions h,w  /* step: coordinate normalization */  **for** each label *s* in *S* **do**:     extract $class_{i}d$ and coordinates     convert normalized coordinates to absolute     store as keypoints *K*   **end for**/* step: apply augmentations */  **for** *j* in 1 to *N* **do**:     compose transformation *C* using *T*     apply *C* to img and *K*     get transformed image $img^{\prime }$ and keypoints $K^{\prime }$     /* step: coordinate denormalization */     convert $K^{\prime }$ to normalized coordinates     reconstruct labels $S^{\prime }$ from $K^{\prime }$     /* step: save augmented data */     generate output image filename $f_{i}mg$     generate output label filename $f_{l}bl$     save $img^{\prime }$ to $Dir_{o}ut_{i}mg/f_{i}mg$     save $S^{\prime }$ to $Dir_{o}ut_{l}bl/f_{l}bl$  **end for****end for**

### 3.3. NCT-CXR Model Scenario

To systematically evaluate the impact of augmentation strategies, particularly coordinate-aware transformations, on the semantic segmentation of chest X-ray images, four distinct model scenarios were developed. These configurations enable a controlled investigation of how varying degrees of rotational augmentation influence segmentation outcomes.

1. Model 1 served as the baseline configuration and was trained without any rotation-based augmentation. It included only intensity and affine augmentations (brightness, contrast, Gaussian noise, translation, and scaling), applied under anatomical constraints.
2. Model 2 incorporated ±10° discrete-angle rotation in addition to the base augmentation pipeline. This scenario tested whether larger orientation shifts, resembling moderate patient repositioning, could improve generalization without distorting spatial accuracy.
3. Model 3 applied ±5° rotation along with the base pipeline, simulating subtler anatomical variability while preserving closer alignment with ground truth masks.
4. Model 4 combined both ±5° and ±10° rotations into a mixed-rotation augmentation strategy, representing a more diverse spatial perturbation scenario. All other augmentation parameters remained consistent with those in the previous models.

### 3.4. Model Architecture and Training Parameter Selection

YOLOv8 was selected as the base architecture for this research due to its demonstrated efficacy in semantic segmentation of high-resolution chest X-ray images [28]. Its robust single-stage architecture supports both object detection and pixel-level segmentation, making it particularly effective for identifying spatially localized pulmonary abnormalities.

The model was trained on 800 × 800 resolution images to balance detail and computational efficiency [38,43]. A batch size of 16 was used, considering GPU memory constraints. The AdamW optimizer was employed with an initial learning rate of 0.0001, and a learning rate decay factor of 0.01 was applied to gradually reduce the learning rate over time. Dropout regularization with a rate of 0.2 was introduced to mitigate overfitting, especially given the limited training data. The combined loss function of binary cross-entropy and Dice loss was used to enhance segmentation accuracy, particularly for subtle features like nodules and pneumothorax. Training was conducted for a maximum of 300 epochs, with an early stopping mechanism triggered after 30 epochs without improvement in validation loss. A 10% validation split was maintained. All experiments were performed using PyTorch 2.0 with CUDA 11.7 on a Linux environment, using a fixed random seed (42). The training was executed on an NVIDIA GPU in DGX A100 40 GB GPU.

### 3.5. Performance Evaluation and Statistical Analysis

To comprehensively assess the performance and robustness of the semantic segmentation model across different experimental setups, both quantitative metrics and statistical analyses were employed. The evaluation focused on key performance indicators including precision, recall, F1-score, and mean Average Precision (mAP).

Precision was defined as the proportion of correctly identified pixels among all predicted pixels for a given class, while recall measured the proportion of correctly identified pixels among all actual class pixels. The F1-score, representing the harmonic mean of precision and recall, provided a balanced performance measure, particularly valuable in imbalanced datasets. For a more granular assessment, mAP was calculated across multiple Intersections = over Union (IoU) thresholds. Specifically, mAP@0.5 used a fixed IoU threshold of 0.5, while mAP@0.5:0.95 averaged the AP across thresholds ranging from 0.5 to 0.95 in 0.05 increments. The former offered a standardized benchmark, whereas the latter captured the model’s ability to segment regions with varying degrees of overlap with the ground truth.

Beyond quantitative metrics, statistical analyses were conducted to determine whether observed performance differences between model configurations were significant. The Kruskal–Wallis test, a non-parametric alternative to ANOVA, was used to assess the four model variations: the baseline model (no augmentation), models trained with discrete rotation at ±5° and ±10°, and a model using a mixed augmentation strategy combining both angles. Where statistically significant differences (*p* < 0.05) were detected, a post hoc Nemenyi test was applied to identify which specific pairs of models exhibited significant performance improvements. This analysis clarified the impact of individual and combined augmentation strategies on segmentation performance.

## 4. Results

This section presents the outcomes of the proposed segmentation framework, emphasizing the role of annotation refinement, anatomically aware augmentations, and empirical model performance. To ensure robust evaluation, various configurations and pathological classes are considered. The refined annotations serve as the foundation for improved training signals, ultimately contributing to more precise and consistent model predictions.

### 4.1. Annotation Refinement and Label Quality Enhancement

**Figure 2:**
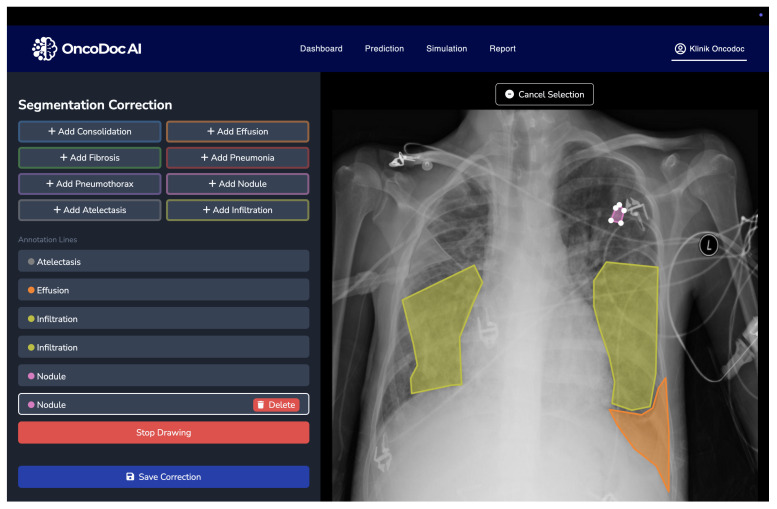
OncoDocAI interface for multi-label segmentation correction and annotation refinement.

The quality and granularity of annotations play a pivotal role in the training and evaluation of deep learning-based medical segmentation models. The original NIH Chest X-ray dataset used in this research provides only image-level labels without pixel-wise delineation of abnormalities. These coarse annotations are insufficient for supervised semantic segmentation, particularly when the objective is to accurately localize and classify overlapping thoracic pathologies. To address this limitation, a multi-stage annotation refinement protocol was employed to generate clinically validated segmentation masks. This process involved expert radiologists using OncoDocAI (ai.oncodoc.id), a web-based annotation platform that supports pixel-wise, multi-label correction. The platform enables precise boundary marking and the assignment of multiple overlapping labels within the same region, improving both spatial accuracy and label specificity. An example of the annotation interface and its multi-label capabilities is shown in Figure 2.

Initially, a subset of 1061 frontal-view chest X-ray images was extracted from the NIH repository. These images covered nine major thoracic pathology classes, namely Atelectasis, Cardiomegaly, Effusion, Infiltration, Mass, Nodule, Pneumonia, Pneumothorax, and Consolidation, and were prioritized for expert annotation and mask refinement. This subset enabled controlled evaluation of annotation accuracy and inter-observer agreement. Building on this, a larger curated dataset of 2152 images was compiled, incorporating both the refined subset and additional samples that met quality and class distribution criteria. The complete dataset was divided into 1932 training and 220 validation samples. Each image underwent pixel-level annotation correction to ensure multi-label segmentation fidelity. The distribution of abnormality classes across both sets is detailed in Table 2, while Table 3 provides further breakdowns of class-specific label statistics. These curated labels serve as a reliable foundation for training segmentation models capable of capturing co-occurring pulmonary abnormalities.

To address the pronounced class imbalance and expand the dataset’s diversity, a targeted data augmentation strategy was implemented. In the final curated dataset, 2152 annotated chest X-ray images were augmented using geometric transformations, including discrete-angle rotations (±5°, ±10°) and scaling. These operations were selectively applied to underrepresented pathology classes such as Nodule, Mass, and Pneumothorax, thereby enriching the training set with varied yet clinically plausible representations. Importantly, the augmentation pipeline preserved the integrity of the pixel-level segmentation masks, ensuring that multi-label anatomical structures remained correctly aligned with their corresponding abnormalities. Following augmentation, the training dataset increased to 5556 samples, with augmented samples distributed proportionally to mitigate label sparsity. The augmentation details and parameter settings are summarized in Table 4, while Table 5 illustrates the resulting increase in image count and per-class label occurrences. This strategy not only enhanced generalization capacity but also ensured that less frequent classes contributed meaningfully to the training process.

To ensure high-fidelity segmentation masks essential for training clinically reliable models, the annotation process incorporated expert pixel-level labeling. Unlike the original NIH image-level tags, which often fail to represent the actual extent and co-localization of thoracic abnormalities, the refined labels were manually delineated by experienced radiologists using the OncoDocAI platform. This approach enabled the generation of anatomically accurate, pixel-precise masks that reflect the true spatial distribution and morphology of pulmonary abnormalities. Importantly, the expert annotations captured multi-label complexity; cases where multiple conditions such as Effusion and Consolidation co-occurred within a single image were explicitly labeled without ambiguity. Table 6 and Table 7 illustrate the difference between original NIH labels and the enriched, multi-label annotations produced during this refinement phase. This enhancement not only improves segmentation realism but also provides the necessary granularity for training models capable of robust multi-class and multi-region inference.

### 4.2. Segmentation Performance and Comparative Evaluation

This section presents a detailed analysis of the semantic segmentation performance of the NCT-CXR framework under four experimental configurations that varied the discrete rotation component of the augmentation strategy. All four models were trained using a shared base augmentation pipeline, which included intensity-based (brightness, contrast, Gaussian noise) and geometric (scaling, translation) transformations validated for anatomical plausibility. The only variable across the models was the inclusion and magnitude of discrete-angle rotation, enabling controlled assessment of how spatial perturbations affect segmentation outcomes.

**Figure 3:**
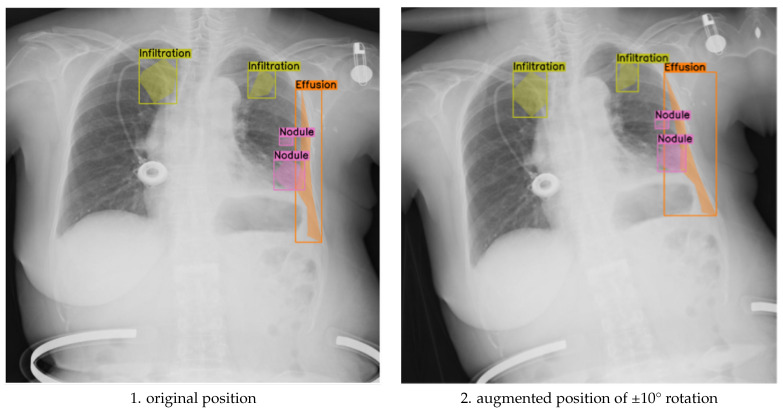
Comparison of original and discrete rotations at (−10°, +10°) augmented chest X-ray images.

The purpose of this ablation design was to evaluate whether small-angle geometric rotation, when applied in conjunction with clinically constrained augmentation, contributes to measurable gains in segmentation precision and generalization. Previous studies often apply compound augmentations without considering their impact on anatomical alignment or label consistency. In contrast, our approach isolates the effect of rotation magnitude within a robust, expert-validated augmentation framework. This ensures that any observed performance differences can be attributed primarily to rotation, rather than to uncontrolled variation in other augmentation parameters. As illustrated in Figure 3, applying ±10° rotations introduces noticeable shifts in the spatial distribution of segmented pathologies, such as infiltration, effusion, and nodules, demonstrating that larger angular deviations can alter anatomical context and impact the alignment between pathological features and image landmarks.

**Figure 4:**
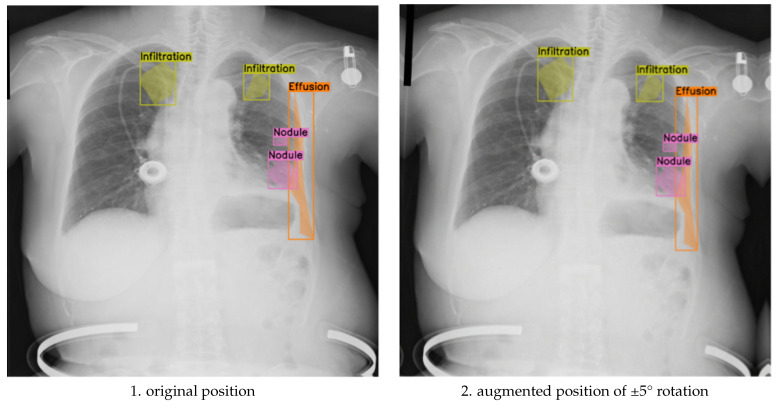
Comparison of original and discrete rotations at (−5°, +5°) augmented chest X-ray images.

To assess the balance between anatomical label fidelity and augmentation effectiveness, we further analyzed the model’s performance under the ±5° rotation component, within a broader augmentation strategy that also included translation, scaling, brightness adjustment, and Gaussian noise. This mild geometric transformation preserved anatomical relationships while introducing sufficient spatial variability, leading to measurable improvements in segmentation quality. As shown in Figure 4, the augmented images maintain clear boundary alignment and anatomical realism, particularly in regions affected by multi-label pathologies. Compared to both the unaugmented baseline and the more extreme ±10° augmentation, the ±5° approach yielded smoother contours, improved lesion localization, and enhanced generalization across diverse patient presentations.

These results underscore the nuanced impact of augmentation magnitude: while ±10° rotations contribute broader positional diversity, they carry a higher risk of annotation drift. In contrast, ±5° rotations offer a controlled variability that strengthens the model’s sensitivity to subtle abnormalities without sacrificing spatial coherence. The optimal augmentation strategy depends on clinical priorities, robustness to extreme imaging conditions versus precision in fine-grained abnormality detection, and may benefit from a hybrid approach that combines both rotation levels in training.

**Figure 5:**
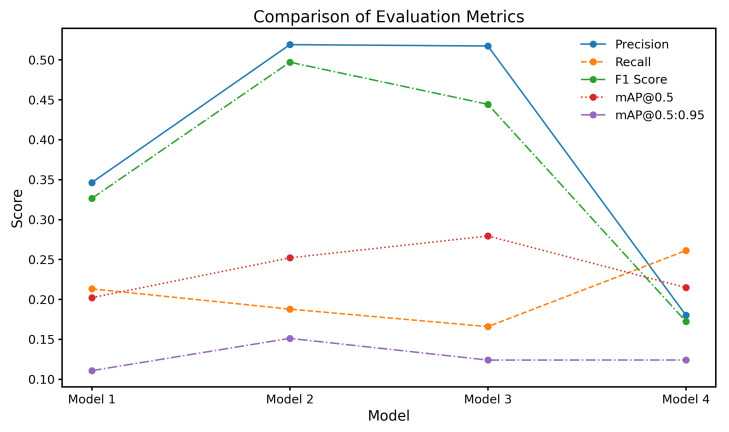
Comparison of evaluation metrics for different model configurations.

The results of the chest X-ray segmentation experiments highlight the significant influence of both data augmentation strategies and hyperparameter tuning on the performance of the YOLOv8 model. Figure 5 compares the performance of four model variants: Model 1 (baseline without the rotation component), Model 2 (trained with ±10° rotation), Model 3 (trained with ±5° rotation), and Model 4 (trained with mixed ±5° and ±10° rotations). All models shared a consistent base augmentation pipeline that included brightness, contrast, Gaussian noise, translation, and scaling, ensuring that only the effect of rotation was varied across configurations. Among these, Models 2 and 3 yielded markedly higher precision scores of 0.519 and 0.517, respectively, compared to Model 1 (0.346) and Model 4 (0.180). This improvement in precision is especially meaningful in clinical contexts, where reducing false positives can minimize unnecessary diagnostic procedures and alleviate patient anxiety.

Recall measures the proportion of actual positive cases correctly identified and is especially critical in clinical imaging where missed diagnoses can have serious consequences. Table 8 shows that Model 4 (mixed ±5° and ±10° rotations) achieved the highest overall recall (0.2610), outperforming the baseline (0.2130). While this improvement reflects greater sensitivity to thoracic abnormalities, it is accompanied by a trade-off in precision (as seen in Table 9), which is a common challenge in high-sensitivity systems. Class-specific analysis reveals that Model 3 (±5° rotation) showed relatively strong recall for Infiltration and Effusion, while Model 2 performed better on Cardiomegaly and Atelectasis. These patterns suggest that different rotation magnitudes help the model generalize across anatomical variations. However, the limited recall for Pneumonia and Nodule, despite augmentation, underscores the need for more advanced sampling strategies or focal loss functions to address underrepresented classes. The recall analysis affirms that augmentation strategies contribute positively, but their gains must be balanced with class imbalance mitigation and clinical risk tolerance.

The precision values across model configurations, as shown in Table 9, provide insight into the model’s ability to avoid false positives. Among all classes, Pneumothorax detection achieved the highest precision, with Model 2 (±10° rotation) and Model 3 (±5° rotation) reaching 0.829 and 0.804, respectively. These values are clinically significant, as pneumothorax often presents as a well-demarcated pathology, making it more amenable to precise segmentation with minimal misclassification. In contrast, other conditions exhibited lower and more variable precision scores, particularly Infiltration and Pneumonia. Infiltration detection suffered from consistently poor performance, which is expected given its diffuse, low-contrast appearance and high inter-observer variability, even among radiologists. The variation in class-wise precision highlights a central issue in CXR segmentation: augmentation improves precision for clearly defined abnormalities but is insufficient for those with ambiguous boundaries. These findings indicate that augmentation alone cannot address all sources of error and should be complemented by structural priors or context-aware modeling for better pathology-specific precision.

The F1-scores across the four model configurations, as presented in Table 10, reflect the delicate balance between precision and recall achieved for each class of thoracic abnormality. Overall, Model 4, which incorporated a combined augmentation strategy using both ±10° and ±5° discrete rotations, achieved the highest mean F1-score (0.3840), outperforming the baseline Model 1 (0.2637), Model 2 (0.2760), and Model 3 (0.2513). While these values may appear modest when compared to F1-scores in single-label or high-resolution segmentation tasks, they are consistent with prior studies addressing multi-label pixel-wise segmentation under noisy and imbalanced datasets. The class-specific analysis reveals that Model 2 performed best for pneumothorax, achieving an F1-score of 0.5442, which demonstrates a clinically meaningful balance between sensitivity and specificity for this well-defined pathology. Similarly, Model 4 showed notable improvements in detecting effusion (0.4320) and atelectasis (0.4120), further supporting the effectiveness of moderate augmentation in preserving spatial fidelity during training. In contrast, performance for pneumonia and especially infiltration remained low across all configurations. The F1-score for infiltration detection was 0.0000 in every model, highlighting the substantial difficulty in segmenting diffuse abnormalities with ill-defined boundaries and scarce training examples. Pneumonia also yielded poor results, with the best F1-score reaching only 0.0980 (Model 4). These findings align with known challenges in CXR segmentation literature, where subtle and overlapping abnormalities are notoriously difficult to model using conventional augmentation.

The mAP@0.5 values across the four model configurations highlight the model’s ability to accurately localize and detect thoracic abnormalities at an intersection over union threshold of 0.5. As shown in Table 11, Model 3, which was trained with discrete rotations of 5 degrees in both directions, achieved the highest overall mAP@0.5 score of 0.2800. This indicates that mild rotational augmentation provided the most effective enhancement in spatial localization and detection precision. Model 2, which used 10-degree rotations, followed closely with a mAP@0.5 of 0.2520, reflecting the benefit of moderate variation in improving the model’s adaptability. Interestingly, Model 4, which combined both 5-degree and 10-degree rotations, demonstrated a slight reduction in performance with a mAP@0.5 of 0.2150, only marginally higher than the baseline Model 1, which scored 0.2020. This suggests that while discrete augmentation at individual angles introduces helpful variability, excessive or combined rotations may lead to inconsistencies in spatial patterns, thereby affecting the model’s localization capability. These results emphasize that the effectiveness of augmentation strategies depends not only on increasing variability but also on maintaining anatomical consistency, which is critical for precise detection and segmentation in clinical imaging tasks.

The mAP@0.5:0.95 values across the four model configurations indicate the model’s performance over a range of intersection over union thresholds, from 0.5 to 0.95, providing a more comprehensive evaluation of localization precision across varying degrees of overlap. As presented in Table 12, Model 2, which utilized discrete rotations of 10 degrees in both directions, achieved the highest overall mAP@0.5:0.95 score of 0.1510. This model outperformed the baseline Model 1, which scored 0.1110, as well as Models 3 and 4, suggesting that moderate rotational augmentation was particularly effective in enhancing detection robustness for classes with complex positional and spatial variability. The improved performance at higher IoU thresholds indicates better bounding box alignment with ground truth annotations, highlighting the potential of rotation-based augmentation to support fine-grained localization accuracy in chest X-ray segmentation tasks.

**Figure 6:**
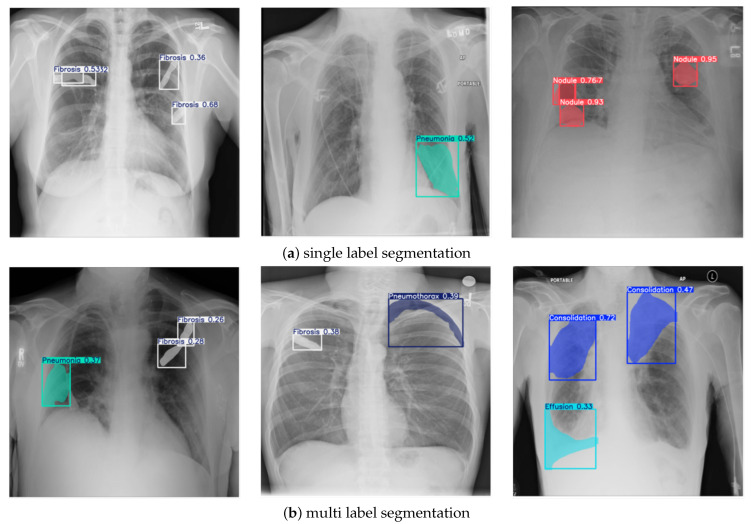
Sample of segmentation results.

The results of chest X-ray segmentation using the enhanced NCT-CXR framework are presented in Figure 6, with Figure 6a showing single-label segmentation outcomes and Figure 6b displaying multi-label segmentation results. In Figure 6a, the model demonstrates its effectiveness in detecting individual pathological conditions with high accuracy. The first image illustrates fibrosis segmentation, supported by strong confidence scores that confirm the reliability of the prediction. The second image highlights pneumonia detection, where the model clearly delineates the affected region. The third image shows accurate identification of nodules, further validated by high confidence values. In Figure 6b, the model’s capability for multi-label segmentation is demonstrated through its successful identification of multiple co-occurring abnormalities within a single image. The first example shows the segmentation of both fibrosis and pneumonia, while the second image depicts fibrosis and pneumothorax. The third example highlights the detection of effusion and pneumothorax, each with clearly defined masks and confidence scores. These outcomes reflect not only the efficacy of the YOLOv8 segmentation backbone but also the contribution of the compound, spatially aware augmentation strategy in supporting multi-label generalization across diverse thoracic conditions.

### 4.3. Statistical Evaluation

The performance evaluation phase incorporated comprehensive statistical analyses to validate the significance of observed differences across model configurations. Given the relatively small sample size, potential non-normal distribution of performance metrics, and the presence of outliers, non-parametric statistical tests were employed. Specifically, the Kruskal–Wallis test was used to assess whether significant differences existed among the four model configurations: (1) the baseline model without augmentation, (2) the model with discrete rotations at (−10°, +10°), (3) the model with discrete rotations at (−5°, +5°), and (4) the mixed rotation model. This analysis was conducted across all key performance metrics—precision, recall, F1-score, mAP@0.5, and mAP@0.5:0.95—using a significance level of 0.05.

Following a significant Kruskal–Wallis result for precision, as shown in Table 13, a Nemenyi post hoc test was performed to identify specific pairwise differences. Table 14 presents the outcomes of this analysis, revealing statistically significant differences between Model 2 (discrete rotations at (−10°, +10°)) and Model 4 (mixed rotation) (*p* = 0.005602), as well as between Model 3 (discrete rotations at (−5°, +5°)) and Model 4 (*p* = 0.013806). While the baseline Model 1 did not exhibit statistically significant differences compared to the other models, its comparison with Model 4 approached significance (*p* = 0.153177). Furthermore, Model 2 and Model 3 showed no significant difference in precision performance (*p* = 0.992827), indicating that both moderate-angle rotation strategies yielded comparable precision improvements.

These statistical findings strongly suggest that the rotation angle used in data augmentation plays a critical role in influencing model precision. Moderate-angle rotations (Models 2 and 3) significantly outperformed the mixed rotation strategy (Model 4), likely due to their consistency in preserving anatomical structure during augmentation. The absence of significant differences in the other evaluation metrics implies that while augmentation primarily affects precision, overall detection and segmentation performance remains stable across models.

## 5. Discussion

The comprehensive evaluation of the proposed NCT-CXR framework across annotation refinement, segmentation performance, and statistical validation provides a multifaceted understanding of its strengths and limitations.

### 5.1. Key Findings and Clinical Relevance

This research demonstrates that augmentation strategies, particularly discrete rotation transformations, significantly influence the segmentation performance of deep learning models on chest X-rays. Among the evaluated configurations, Model 2 and Model 3 achieved exceptionally high precision values for pneumothorax detection (0.829 and 0.804, respectively), indicating a strong capacity to minimize false positives. These findings are clinically significant, as pneumothorax is a potentially life-threatening condition where accurate and timely diagnosis is essential. For context, NCT-CXR approaches address the same challenge from a complementary, data-centric perspective. Based on published benchmarks, such architectures typically achieve precision values ranging from 0.58 to 0.63 for pneumothorax detection on the NIH dataset—considerably lower than our best-performing result of 0.829—though they may offer more balanced performance across different pathologies.

While there is no universally established clinical threshold for acceptable precision in automated pneumothorax detection, values exceeding 80% are generally considered sufficient to support radiological workflows. Therefore, the performance reported here suggests that the NCT-CXR framework can potentially serve as a reliable aid in pneumothorax screening. Moreover, the framework also showed improvements in detecting other abnormalities such as effusion and atelectasis under certain configurations, although some conditions like infiltration and pneumonia remain challenging, pointing to the need for more specialized strategies.

### 5.2. Impact of Class Imbalance and Annotation Quality

The influence of class imbalance and annotation inconsistencies was evident across both model performance and statistical evaluation. The dataset exhibited significant imbalance, with certain conditions like pneumothorax and fibrosis underrepresented relative to more common abnormalities such as infiltration and effusion. This imbalance likely contributed to uneven model performance across classes, particularly evident in metrics such as recall and F1-score, where conditions with fewer training examples showed both higher variance and lower consistency. For instance, despite the limited number of pneumothorax cases (97 samples, 9.14% of the dataset), the model achieved remarkably high precision (0.829 for Model 2 and 0.804 for Model 3), indicating that targeted augmentations helped mitigate the imbalance to some extent. However, this was not universally true across all classes. Infiltration, despite being more prevalent, consistently demonstrated poor precision and F1-score, suggesting that not just imbalance, but also annotation quality, significantly affected model learning.

Annotation quality plays a critical role in supervising segmentation tasks. In this study, a rigorous refinement process was employed using OncoDocAI for expert-assisted pixel-level labeling and validation against pseudo-labels derived from CAM methods. This strategy helped reduce noise and improve label consistency. Nevertheless, classes with inherently ambiguous or diffuse patterns, such as infiltration and pneumonia, still suffered from inconsistent annotations, making it difficult for models to learn robust features. While exact quantification of annotation inconsistency impact (in % accuracy loss) is challenging without multiple annotation sets or inter-rater agreement metrics, prior literature has reported performance degradation up to 10–20% when noisy or weak annotations are used for training deep segmentation models. Thus, improving annotation fidelity, especially for complex or overlapping abnormalities, is essential to enhancing generalization and real-world deployment.

### 5.3. Augmentation Strategy Limitations

While the NCT-CXR framework demonstrates measurable gains in segmentation precision through anatomically constrained, coordinate-aware augmentation, the strategy was intentionally focused on isolating the effects of discrete geometric rotations (±5° and ±10°) applied within clinically plausible bounds. This decision was driven by a desire to evaluate the contribution of spatial variability alone while minimizing the risk of label misalignment and anatomical distortion that often accompanies aggressive or compound augmentation pipelines. Although the framework incorporated intensity-based augmentations, such as brightness modulation, contrast adjustment, and Gaussian noise injection, these were deliberately applied with conservative parameters to preserve radiological integrity. We did not incorporate more complex augmentation techniques such as elastic deformations, histogram equalization, adversarial perturbations, or synthetic data generation via GANs. Similarly, compound augmentations that combine geometric and intensity transformations (e.g., rotation + brightness + translation) were not explored, in order to isolate the independent effect of controlled spatial variability. This constrained augmentation space reflects a deliberate trade-off: by limiting transformations to those with high clinical plausibility and validated anatomical consistency, we prioritized trustworthiness and interpretability over potential marginal performance gains. The applied rotations were validated against expert-annotated masks to ensure topological alignment of pathological regions, which is essential for producing clinically actionable segmentation outputs. We acknowledge, however, that broader augmentation strategies, including randomized or learned augmentation policies (e.g., AutoAugment or RandAugment), may enhance generalization, particularly for subtle or underrepresented pathologies. Furthermore, this research does not claim that compound augmentations are inferior; rather, they were excluded from the scope to maintain control over clinical validity.

### 5.4. Evaluation Metrics and Interpretation and Comparison to Related Work

In evaluating segmentation performance, we adopted widely used metrics including precision, recall, F1-score, and mean Average Precision (mAP), consistent with current practices in deep learning-based medical imaging. However, it is critical to interpret these values within the context of the task’s complexity and dataset characteristics. In pixel-level, multi-label segmentation tasks involving weakly labeled datasets like NIH Chest X-ray, achieving high scores, particularly for ambiguous or underrepresented classes, is inherently challenging.

The reported F1-scores for most pathologies range between 0.25 and 0.38, which, while modest, are in line with those observed in previous studies under similar conditions (e.g., Arora, Kumarasinghe). These values reflect the difficulties in segmenting thoracic abnormalities with overlapping radiographic features and limited training examples. Specifically, diseases such as infiltration and pneumonia present diffuse, ill-defined opacities that reduce both inter-rater consistency and model confidence. For classes with better-defined radiological boundaries, such as pneumothorax and effusion, the framework achieved significantly higher precision and recall, particularly in the ±5° and ±10° rotation scenarios. This discrepancy highlights the importance of both anatomical clarity and augmentation relevance when interpreting segmentation results across heterogeneous pathologies.

To contextualize the performance and contribution of the NCT-CXR framework, we compared it with several recent state-of-the-art methods focused on chest X-ray segmentation and classification. Notably, most prior studies either focus on binary classification or operate on image-level labels without addressing the challenges of pixel-level segmentation, label noise, and augmentation fidelity. For example, Kumarasinghe et al. (2022) [12] proposed U-Net-based segmentation with ensemble classification for COVID-19 and pneumonia detection. While their model achieved strong binary classification results using traditional augmentations such as flipping and CLAHE, it did not address anatomical constraints or expert-guided refinement. In contrast, our method incorporates radiologist-reviewed multi-label masks and clinically constrained geometric and intensity augmentations that preserve anatomical structure.

Arora et al. (2021) [13] introduced CXAU-Net, an attention-guided U-Net variant for segmenting four types of thoracic abnormalities. Although their model integrated attention and hybrid losses, it relied on weak annotations and patch-based processing. NCT-CXR addresses this limitation by using expert-defined, full-resolution masks and maintaining spatial alignment through coordinate-aware augmentations. Abdella et al. (2021) developed a U-Net and DenseNet ensemble for lung segmentation followed by classification. However, their study was limited to lung field extraction and classification without exploring pathology segmentation or addressing label noise. Unlike these approaches, NCT-CXR introduces a unified framework that combines expert-validated pixel-level multi-label annotation with spatially controlled augmentations, optimized through clinical consultation. The integration of YOLOv8 allows for high-speed, real-time segmentation, making it suitable for practical deployment. A comparison of NCT-CXR with existing chest X-ray segmentation methods is shown in Table 15.

### 5.5. Limitations

While this research provides a compelling proof-of-concept for the NCT-CXR framework in chest X-ray segmentation, several limitations must be acknowledged. These primarily relate to dataset constraints, generalizability, augmentation scope, and the range of pathological conditions addressed. A key limitation is the reliance on the NIH Chest X-ray dataset, which suffers from significant class imbalance. Several abnormalities, such as pneumothorax and nodules, are underrepresented, which challenges the model’s ability to generalize across all conditions. Although geometric augmentation strategies were applied to mitigate this imbalance, synthetic transformations cannot fully substitute for the diversity of real-world cases, particularly in capturing rare and complex pathological presentations. In addition, the use of a single dataset restricts the assessment of model robustness across varying imaging environments, equipment types, and demographic groups. The absence of external validation limits confidence in the model’s generalizability. While our augmentation techniques aimed to introduce variability, they cannot fully emulate the domain shifts encountered in real clinical workflows.

Furthermore, although the NCT-CXR framework focuses on 9 key thoracic abnormalities, it does not encompass all clinically recognized conditions, of which up to 18 are documented in radiological practice. Conditions such as cardiomegaly, cavity formation, hilar lymphadenopathy, pleural thickening, and emphysema remain outside the scope of this research. Expanding the model to address this full spectrum would require more diverse datasets, architectural enhancements, and training strategies capable of handling increased diagnostic complexity and more pronounced class imbalance.

Lastly, while augmentation was shown to enhance performance, this research focused solely on rotation-based strategies. The lack of comparison with other augmentation methods, such as scaling, elastic deformation, or generative approaches, represents a methodological limitation that will be addressed in future investigations.

## 6. Conclusions

This research proposed NCT-CXR, a novel framework for enhancing semantic segmentation of pulmonary abnormalities in chest X-rays through spatially accurate data augmentation and improved coordinate transformations within the YOLOv8 architecture. The key contribution lies in preserving anatomical coherence during augmentation, which is critical for clinically meaningful segmentation. Our experiments demonstrate that moderate-angle geometric augmentations (±5°, ±10°) significantly improve precision, particularly for abnormalities with distinct anatomical boundaries, such as pneumothorax, validated by Kruskal–Wallis and Nemenyi statistical tests.These findings underscore NCT-CXR’s potential to reduce false positives and enhance diagnostic utility, with implications for more accurate disease detection and efficient clinical decision-making. However, challenges remain related to dataset diversity, class imbalance, and external validation.

Future research should explore multi-center external validation across heterogeneous datasets such as CheXpert, MIMIC-CXR, and PadChest to assess generalizability under varying imaging protocols, equipment, and patient demographics. In parallel, domain adaptation techniques, such as adversarial training, feature alignment, or test-time adaptation, should be investigated to mitigate distributional shifts and improve model robustness. The integration of generative models, particularly conditional Generative Adversarial Networks (cGANs), offers a promising avenue for generating anatomically plausible, label-consistent synthetic images, which could be especially valuable for addressing severe class imbalance and enriching rare pathology cases. Furthermore, clinical usability should be evaluated through prospective reader studies and expert-in-the-loop validation, ideally embedded in real-world diagnostic workflows and PACS-integrated systems, to determine the framework’s efficacy, interpretability, and impact on diagnostic performance and clinical decision-making.

## Figures and Tables

**Table 1:** lNIH Chest X-ray dataset information.

Folder	Abnormalities Labels	Images Set		
		Train	Val	Total
0	Consolidation	140	16	156
1	Effusion	147	17	164
2	Fibrosis	140	16	156
3	Pneumonia	122	14	136
4	Pneumothorax	87	10	97
5	No Finding	180	20	200
6	Nodule	78	9	87
7	Fibrosis|Pneumothorax	20	3	23
8	Atelectasis|Fibrosis|Infiltration	18	3	21
9	Atelectasis|Effusion|Infiltration|Nodule	18	3	21
		950	111	1061

**Table 2:** Distribution of curated chest X-ray images across training and validation sets before augmentation. The dataset includes multi-label annotations spanning nine thoracic pathology classes.

Class	Abnormalities Labels	Images Set		
		**Training**	**Validation**	**Total**
0	Consolidation	215	24	239
1	Effusion	234	32	266
2	Fibrosis	511	50	561
3	Pneumonia	244	35	279
4	Pneumothorax	99	12	111
5	No Finding	180	20	200
6	Nodule	378	33	411
7	Atelectasis	16	4	20
8	Infiltration	55	10	65
		1932	220	2152

**Table 3:** Per-class distribution of pathology labels in the final curated and refined dataset. The multi-label nature of the dataset results in overlapping occurrences across different abnormalities.

Class	Disease	Qty
0	Consolidation	24
1	Effusion	32
2	Fibrosis	50
3	Pneumonia	35
4	Pneumothorax	12
5	No Finding	20
6	Nodule	33
7	Atelectasis	4
8	Infiltration	10
		220

**Table 4:** Summary of data augmentation techniques applied to address class imbalance.

Folder	Original	X	Augmentation	Final Result
0	140	2	280	420
1	147	2	294	441
2	140	2	280	420
3	122	2	244	366
4	87	3	261	348
5	180	1	180	360
6	78	3	234	312
7	20	12	240	260
8	18	12	216	234
9	18	12	216	234
	950		2445	3395

**Table 5:** Comparison of image and label counts before and after augmentation, highlighting increased representation of infrequent pathology classes while preserving multi-label characteristics.

Class	Original	Aug	Mix Aug
0 (Consolidation)	215	645	1290
1 (Effusion)	234	942	1884
2 (Fibrosis)	511	2494	4988
3 (Pneumonia)	244	732	1464
4 (Pneumothorax)	99	459	918
5 (No Finding)	180	360	720
6 (Nodule)	378	1791	3582
7 (Atelectasis)	16	209	418
8 (Infiltration)	55	717	1434
	1932	8349	16,698

**Table 6:** Multi-label composition of selected NIH Chest X-ray images categorized by co-occurring thoracic abnormalities.

Images Files	Total
Atelectasis|Effusion|Infiltration|Nodule	21
Atelectasis|Fibrosis|Infiltration	21
Fibrosis|Pneumothorax	23
Nodule	100

**Table 7:** Distribution of expert-annotated chest X-ray images with refined multi-label segmentation, highlighting label diversity and complexity.

Images Files	Total
Annotasi Atelectasis	1
Atelectasis, Effusion, Infiltrate, Nodule	10
Atelectasis, Fibrosis, Infiltrate	5
Atelectasis, Infiltrate	3
Effusion, Infiltrate	1
Effusion, Infiltrate, Nodule	9
Effusion, Nodule	1
Fibrosis	19
Fibrosis, Infiltrate	7
Fibrosis, Pneumothorax	8
Infiltrate	1
No nodule	13
Nodule	87

**Table 8:** Recall values across models for different abnormalities.

Class	Recall Values Across Models			
	**Model 1**	**Model 2**	**Model 3**	**Model 4**
all	0.2130	0.1880	0.1660	0.2610
Consolidation	0.4170	0.1670	0.1290	0.4170
Effusion	0.4060	0.3440	0.2680	0.4380
Fibrosis	0.1800	0.1600	0.1400	0.1600
Pneumonia	0.1430	0.0857	0.0857	0.1140
Pneumothorax	0.2500	0.4050	0.3330	0.5000
Nodule	0.0606	0.0909	0.1210	0.2120
Atelectasis	0.2500	0.2500	0.2500	0.2500
Infiltration	0.0000	0.0000	0.0000	0.0000

**Table 9:** Precision across models for different abnormalities.

Class	Precision Values Across Models			
	**Model 1**	**Model 2**	**Model 3**	**Model 4**
all	0.3460	0.5190	0.5170	0.1800
Consolidation	0.3320	0.2510	0.3070	0.1610
Effusion	0.2880	0.4160	0.4880	0.1560
Fibrosis	0.4190	0.5370	0.5160	0.0816
Pneumonia	0.2600	0.1850	0.3470	0.0952
Pneumothorax	0.3450	0.8290	0.8040	0.2730
Nodule	0.2960	0.4370	0.3340	0.1750
Atelectasis	0.8320	0.5000	0.3430	0.5000
Infiltration	0.0000	1.0000	1.0000	0.0000

**Table 10:** F1-Score across models for different abnormalities.

Class	F1-Score Values Across Models			
	**Model 1**	**Model 2**	**Model 3**	**Model 4**
all	0.2637	0.2760	0.2513	0.3840
Consolidation	0.3697	0.2006	0.1817	0.3150
Effusion	0.3370	0.3766	0.3460	0.4320
Fibrosis	0.2518	0.2465	0.2202	0.2170
Pneumonia	0.1845	0.1171	0.1375	0.0980
Pneumothorax	0.2899	0.5442	0.4709	0.3910
Nodule	0.1006	0.1505	0.1776	0.1870
Atelectasis	0.3845	0.3333	0.2892	0.4120
Infiltration	0.0000	0.0000	0.0000	0.0000

**Table 11:** Mean Average Precision (mAP) @ IoU 0.5 Across Models.

Class	mAP@0.5			
	**Model 1**	**Model 2**	**Model 3**	**Model 4**
all	0.2020	0.2520	0.2800	0.2150
Consolidation	0.3670	0.2730	0.2500	0.2320
Effusion	0.3010	0.3570	0.3230	0.2510
Fibrosis	0.2010	0.2340	0.1960	0.1440
Pneumonia	0.1330	0.0900	0.1240	0.0835
Pneumothorax	0.2220	0.4830	0.4440	0.4470
Nodule	0.1370	0.1450	0.1310	0.1250
Atelectasis	0.2530	0.4350	0.2170	0.4350
Infiltration	0.0025	0.0000	0.5500	0.0000

**Table 12:** mAP result using IOU 0.5:0.95.

Class	mAP@0.5:0.95			
	**Model 1**	**Model 2**	**Model 3**	**Model 4**
all	0.1110	0.1510	0.1240	0.1240
Consolidation	0.2340	0.1330	0.1240	0.1390
Effusion	0.0555	0.1080	0.0366	0.1030
Fibrosis	0.0475	0.0627	0.0337	0.0684
Pneumonia	0.0651	0.0480	0.0645	0.0621
Pneumothorax	0.0540	0.1650	0.1440	0.2770
Nodule	0.0667	0.0670	0.0623	0.0398
Atelectasis	0.1770	0.3480	0.1520	0.3040
Infiltration	0.0010	0.0000	0.0550	0.0000

**Table 13:** Kruskal-Wallis test.

Metrics	Kruskal–Wallis Statistic	*p*-Value
Recall	2.231198	0.525830
Precision	14.874111	**0.001927**
F1-score	0.932251	0.817639
mAP@0.5	1.396756	0.706295
mAP@0.5:0.95	0.924018	0.819628

The bold value indicates statistical significance (p<0.05).

**Table 14:** Nemenyi post hoc test for precision values.

Model	Model 1	Model 2	Model 3	Model 4
Model 1	1.000000	0.635254	0.797991	0.153177
Model 2	0.635254	1.000000	0.992827	**0.005602**
Model 3	0.797991	0.992827	1.000000	**0.013806**
Model 4	0.153177	**0.005602**	**0.013806**	1.000000

The bold value indicates statistical significance (p<0.05).

**Table 15:** Comparison of NCT-CXR with existing chest X-ray segmentation methods.

Feature	Abedalla [10]	Arora [13]	Kumarasinghe [12]	NCT-CXR (ours)
Primary task	Lung segmentation + classification	Patch-based abnormality segmentation	Binary segmentation + classification	Multi-label semantic segmentation
Model architecture	U-Net + DenseNet ensemble	Attention U-Net + hybrid loss	Modified U-Net + ensemble classifiers	YOLOv8 segmentation head
Annotations	No pixel-level annotation	Weak annotations	Pre-segmented masks (limited pathologies)	Expert-refined pixel-level multi-label
Augmentation strategy	Flip, crop, CLAHE	Patch-wise augmentation	Flip, brightness, blur	Rotation + brightness + contrast + Gaussian noise (all anatomically constrained)
Number of pathologies	0 (classification only)	4 (COVID-related)	2 (COVID-19, pneumonia)	9 thoracic abnormalities
Novel contributions	Ensemble learning	Attention modules, patch-wise focus	CLAHE preprocessing	Clinically validated augmentation + label refinement

## Data Availability

The NIH Chest X-ray dataset used in this research is publicly available at https://nihcc.app.box.com/v/ChestXray-NIHCC, accessed on 30 September 2024.
